# The Use of Telemedicine and Other Strategies by Registered Dietitians for the Medical Nutrition Therapy of Patients With Inherited Metabolic Disorders During the COVID-19 Pandemic

**DOI:** 10.3389/fnut.2021.637868

**Published:** 2021-04-27

**Authors:** Rani H. Singh, Theresa Pringle, Aileen Kenneson

**Affiliations:** ^1^Department of Human Genetics, Emory University, Atlanta, GA, United States; ^2^Department of Pediatrics, Emory University, Atlanta, GA, United States

**Keywords:** medical nutrition therapy, inherited metabolic disorders, telemedicine, COVID-19, registered dietitian

## Abstract

The clinical management of patients with inherited metabolic disorders (IMDs) includes medical nutrition therapy (MNT) by a registered dietitian (RD). We utilized an online quantitative and qualitative survey to characterize the practices of RDs treating patients with IMDs during the COVID-19 pandemic and to identify challenges and unmet needs. We received responses from 117 RDs. Results indicate that RDs are using alternate methods to engage this vulnerable population and provide MNT during the pandemic, including offering telemedicine appointments. Barriers to implementation of telemedicine include the limitations of virtual visits (inability to conduct physical exams and collect blood samples), time, patient knowledge of technology, audio problems, and patient access to internet, computers, or smartphones. RDs have addressed these barriers by extending prescriptions without a medical exam, relying on local facilities for blood draws, increasing the number of patients that use at-home filter papers for blood monitoring, and expanding the use of phone calls and emails. RDs identified patient education materials to facilitate telemedicine visits as a primary unmet need. Despite the reported barriers and limitations of telemedicine for MNT of IMDs, there was widespread satisfaction with the approach among RDs, with 96.9% reporting that they were somewhat or very satisfied with telemedicine. Although this survey focused on barriers, benefits of telemedicine for both RDs and patients were also reported. Identification of barriers and unmet needs can help clinics plan strategies to maximize telemedicine delivery models, to improve efficiency and patient outcomes, and to support sustained use of telemedicine post-pandemic.

## Introduction

The COVID-19 pandemic is an unprecedented global public health emergency. One of the major strategies to decrease transmission of the SARS-CoV-2 virus is the implementation of social distancing, and in some cases community stay-at-home orders. Individuals with chronic conditions who need continued healthcare may struggle to maintain continuity of care during this time. Patients with inherited metabolic disorders (IMDs) are included in this category and require regular follow-up to maintain optimal metabolic control.

IMDs are a group of disorders primarily identified through population-based newborn screening programs. IMDs are characterized by deficiency in the ability to metabolize a particular component of proteins, carbohydrates, or fats. Phenotypes vary by disorder and are due to a build-up of the substrate that cannot be metabolized, and/or lack of the end product of the metabolism of said substrate. IMDs require lifelong management and treatment, often focused on the restriction of the offending substrate from the diet, replacement of nutrients with supplements (medical foods), and medications (1, 2). Thus, registered dietitians (RDs) who have received training as genetic metabolic dietitians play an important role in providing medical nutrition management (MNT) for patients as part of the clinical team at metabolic clinics.

Any disruptions to care of patients with IMDs, including lack of access to medical foods or MNT, can lead to cognitive impairment, coma, or death, depending on the IMD. Furthermore, some IMDs are associated with metabolic decompensation in response to illness (3), which can lead to permanent brain damage or death. Indeed, two cases of decompensation and death due to COVID-19 in patients with IMDs have been reported (4). Thus, it is critical that patients with IMDs continue to receive MNT, medical foods, and access to their metabolic team, while limiting risk of exposure to the SARS-CoV-2 virus (5). However, one study reported a 72% reduction in the number of patients whose samples were sent for laboratory analysis and a 59% reduction in the number of patients receiving nutritional support due to changes in care associated with the pandemic (4).

Telemedicine is a potential strategy to improve access to care for patients with IMDs during the COVID-19 pandemic and after (4–6). The specific details of telemedicine visits for MNT for IMDs depend on the disorder, disease severity, and the patient's age and developmental status, as well as local clinic practices. In general, a typical telemedicine MNT visit includes informed consent from the patient and/or guardian, referral information as necessary, and relevant medical history. Recent anthropometrics, ideally collected by the patient's local primary care physician or other healthcare provider, are also reviewed. Laboratory tests that need to be ordered are either collected prior to the appointment through the patient's local healthcare provider or laboratory or are ordered locally after the appointment. If labs were collected and results are available prior to the nutrition telemedicine visit, the results are reviewed with the patient and/or guardian. The RD then conducts a thorough nutrition assessment to better understand the patient's general well-being, social history, and dietary habits. The RD also inquires about current medications, recent illnesses, hospitalizations, and emergency department visits. The RD reviews formula mixing techniques and assesses dietary adherence. If the RD recognizes areas requiring improvement to achieve better metabolic control, he or she provides the necessary nutrition education and counseling to improve areas of poor adherence and lack of understanding. If relevant educational resources (charts, diagrams, videos, etc.) are readily available, the RD also may share his or her screen for the patient and/or guardian to review during the nutrition counseling session. The RD assesses the patient and/or guardian's understanding of the new information and additional nutrition education may be offered for improved compliance. Once the RD has completed the nutrition assessment, the nutrition intervention including the patient's new formula/diet prescription is communicated to the patient and/or guardian. The RD concludes the visit by reviewing the final plan, monitoring frequency, and nutrition goals. A final written plan is provided to the patient and/or guardian. An evaluation component may be included after telemedicine visits to collect feedback from the patient and/or guardian for program evaluation.

Previously-reported barriers to the implementation of telemedicine by metabolic clinics include cost of the technology, availability of technology support, staff lack of familiarity with technology, licensing, credentialing, reimbursement, privacy, and confidentiality (7). Barriers to patient use of telemedicine include preference for in-person appointment, concerns about data security, discomfort with technology, and lack of access to technology and internet service (8–10).

We conducted a cross-sectional survey of RDs managing patients with IMDs during the COVID-19 pandemic. The aim of this survey was to characterize management strategies in the time of COVID-19, identify challenges in implementing telemedicine for managing patients with IMDs, and discuss potential solutions. Identification of barriers and successes can inform the feasibility of creating and/or sustaining telemedicine programs post-pandemic.

## Materials and Methods

We developed a 37-item survey that was distributed online via the Survey Monkey website. The survey consisted of questions about the respondent, their practice, their use of telemedicine for direct patient care, barriers to telemedicine, billing and reimbursement related to telemedicine, and institutional emergency preparedness. Eligible participants were RDs providing MNT for patients with IMDs.

Participants were recruited through the Emory University GNO Metab listserv, which includes all Genetic Metabolic Dietitians International group members as well as other practitioners who treat patients with IMDs or who have an interest in the nutrition management of IMDs globally. The listserv included 453 members at the time of the request to participate; however, all members did not meet the eligibility criteria, as the listserv includes other practitioners (e.g., MDs, diet technicians), as well as RDs who are not involved in direct patient care (e.g., those involved in solely in research). Eligibility criteria were outlined in the recruitment email. Survey responses were collected from April 30 through May 13, 2020. Study participants were offered a $20 gift card to reimburse them for their time and effort. This study was determined to be exempt for approval by the Emory University Institutional Review Board.

Qualitative data for analysis were obtained from open-ended questions on the survey and analyzed using inductive thematic analysis and an iterative process. Two researchers (TP/AK) independently reviewed the open text and identified themes using thematic analysis (11). The two sets of proposed themes were compared and a consensus codebook was developed by the three authors. TP and AK independently assigned the open-ended responses to the themes. Assignments were compared, discrepancies were identified and discussed, and consensus was reached on all assignments.

## Results

Completed surveys were received from 117 RDs who currently provide MNT for patients with IMDs, for a response rate of 25.8%. Ninety-seven (82.9%) were current members of GMDI. Ninety-eight (83.8%) were from the United States, representing 37 states plus Washington DC and one territory. Twelve RDs (10.2%) were from Canada and seven were from other countries. Characteristics of the 117 survey respondents are presented in Table 1.

**Table 1:** Characteristics of RDs who provide nutritional management for patients with IMDs (*N* = 117).

**Question**	**Responses**	**Frequency (%)**
Are you a member of GMDI?	Yes	97 (82.9%)
	No	20 (17.1%)
In which country are you located?	US	98 (83.8%)
	Other	19 (16.2%)
What telemedicine services, if any, does your institution provide for the care of patients with inherited metabolic disorders? (check all that apply) *(10 missing responses)*	None	2 (1.9%)
	I don't know	0 (0%)
	Direct Outpatient care via videoconference	99 (92.5%)
	Direct inpatient care via videoconference	24 (22.4%)
	Consultation with other providers	30 (28.0%)
	Store-and-forward	0 (0%)
	Other	18 (16.8%)
Approximately how many patients with IMDs do you treat? *(11 missing responses)*	Mean	215
	Standard deviation	191
	Range	7–1,000
Have you received training on telemedicine for direct patient care? *(10 missing responses)*	Yes, prior to the COVID-19 outbreak	33 (30.8%)
	Yes, since the COVID-19 outbreak	49 (45.8%)
	No, but I have plans to receive training	10 (9.3%)
	No, and I have no plans to receive training	14 (13.1%)
Does your institution provide telemedicine services? *(10 missing responses)*	Yes	100 (93.4%)
	No	7 (6.5%)
	I don't know	0 (0%)
Are there barriers to full implementation of telemedicine for patients with IMDs in your clinic? *(10 missing responses)*	Yes	76 (71.0%)
	No	31 (29.0%)
How would you describe your telemedicine activities for providing nutrition management to patients with IMDs? *(12 missing responses)*	No plan for development of telemedicine	4 (3.8%)
	Actively planning to implement telemedicine	5 (4.8%)
	Started using telemedicine prior to the COVID-19 pandemic	28 (26.7%)
	Started using telemedicine since the onset of the COVID-19 pandemic	68 (64.8%)
Do you have any of the following needs for telemedicine implementation? (Check all that apply) *(39 did not select a need)*	Training	34 (43.6%)
	Patient education materials	62 (79.5%)
	Provider education materials	31 (39.7%)
	Other	6 (7.7%)
Prior to the COVID-19 outbreak, did your institution have a plan in place for the use of telemedicine during a natural disaster or other public health emergency? *(14 missing responses)*	Yes	13 (12.6%)
	No	58 (56.3%)
	I don't know	32 (31.1%)
Prior to the COVID-19 outbreak, did your institution have plans in place for blood draws during a natural disaster or other public health emergency? *(14 missing responses)*	Yes	6 (5.8%)
	No	55 (53.4%)
	I don't know	42 (40.8%)
Prior to the COVID-19 outbreak, did your institution have plans in place for the provision of medical foods during a natural disaster or other public health emergency? *(14 missing responses)*	Yes	17 (16.5%)
	No	68 (66.0%)
	I don't know	18 (17.5%)

### Barriers to Telemedicine Implementation

Seventy-six RDs (71.0%) reported that there were barriers to the full implementation of telemedicine for patients with IMDs at their institution, including 73 RDs who listed barriers in the open-ended question in the survey. Barriers were also mentioned by 16 RDs in the additional comments at the end of the survey. The major themes related to barriers to telemedicine implementation for MNT for patients with IMDs, along with the number of times each theme was mentioned, are:

#### Limitations of Virtual Visits (*n* = 76)

There are limitations to what can be accomplished during a virtual visit. There is an inability to obtain blood samples for laboratory assessments and some patients are hesitant to go to a local laboratory for blood draws. In addition, it is difficult to obtain accurate anthropometric measurements for prescription maintenance. Some platforms do not allow for multiple providers (including interpreters) to attend the virtual visit concurrently. It can be difficult to read body language or to make a personal connection with the patient, and there may be distractions in the home environment. It also can be difficult to teach hands-on topics such as medical food preparation.

#### Patient Access (*n* = 49)

Many patients lack access to internet, computers with cameras and audio capabilities, or smartphones. Access to necessary software or applications can also be a barrier.

#### Technology Issues (*n* = 26)

Other technology barriers include the availability of a telemedicine platform, audio and video quality, internet connection problems, and provider difficulty with using the telemedicine platform.

#### Patient Preferences (*n* = 19)

Some patients prefer in-person meetings or have religious reasons for not using technology, and language barriers preclude some patients from being able to follow the directions for accessing the telemedicine visit.

#### Patient Knowledge (*n* = 15)

Some patients or families have difficulty utilizing the technology for telemedicine visits. They may lack knowledge about the technology required or the telemedicine platform may be difficult for patients to navigate.

#### Time-Consuming (*n* = 15)

Visit preparation for providers and patients, as well as scheduling, is time-consuming. The process can be cumbersome for families, who may have to complete informed consent and other pre-visit forms. There is limited clinic staff to meet the increased needs related to the coordination of telemedicine visits.

#### Insurance Coverage and Billing Issues (*n* = 14)

There are insurance barriers to seeing out-of-state patients, as well as limitations to bill for telemedicine visits and inability of RDs to bill for services.

#### Institutional Resources and Policies (*n* = 9)

Barriers include the lack of availability of a telemedicine platform within the institution, policies that prohibit telemedicine visits for new patients, policies prohibiting non-MD providers from using telemedicine, limited number of telemedicine platform licenses for the clinic, and institutional selection of a telemedicine platform that does not meet provider needs. Inability to access clinic records while working from home was also mentioned as a barrier.

### Telemedicine Implementation

**Figure 1:**
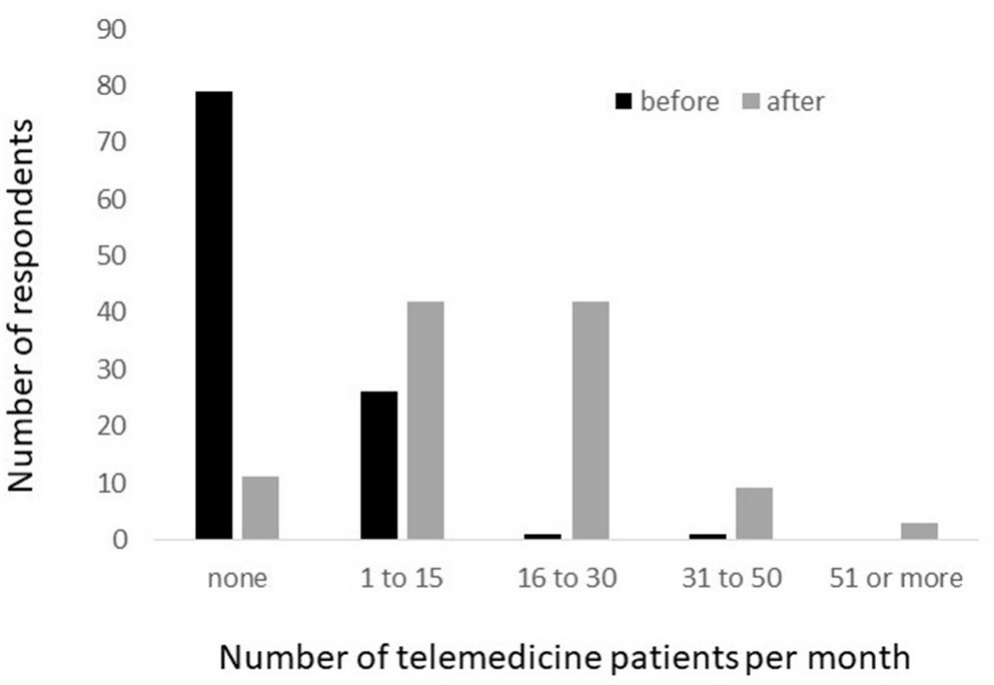
Number of patients with IMDs seen per month via telemedicine, before and after the onset of the COVID-19 pandemic.

Data were available for 107 respondents regarding the use of telemedicine before and after the onset of the pandemic. Respondents indicated that prior to the pandemic, 28 (26.2%) were using telemedicine for patients with IMDs, with most (*n* = 26) seeing fewer than 15 patients per month (Figure 1).

After the onset of the pandemic, 96 RDs reported that they provided telemedicine visits for MNT for patients with IMDs. Four RDs indicated no plans for the implementation of telemedicine for MNT for patients with IMDs, including one RD from the United States and three from other countries. All four indicated that they were consulting with patients via telephone. Barriers to the implementation of telemedicine identified by these four RDs included lack of hospital resources, lack of patient access to technology, and difficulty conducting physical exam and obtaining blood samples when using telemedicine. In addition, seven RDs reported that their institution does not provide telemedicine services for any patient. Overall, the number of patients seen via telemedicine increased greatly in response to the pandemic (Figure 1).

Characteristics of the 96 respondents who were using telemedicine to provide MNT for patients with IMDs are shown in Table 2. The types of patients seen via telemedicine included patients with positive results on newborn screening (45.8%), newly diagnosed patients other than newborn screening (40.6%), hospitalized patients seeking nutrition counseling (11.4%), follow-up patients (99.0%), and research participants (10.4%). However, approximately two-thirds of RDs (68.4%) reported that there are patient populations that cannot be seen via telemedicine, including newly diagnosed patients (45.5%), patients in hospitals (33.3%), out-of-state patients (22.7%), and other (54.5%). The other types of patients that cannot be seen via telemedicine as described by participants in an open-ended question included patients without access to technology (*n* = 19), patients with language barriers (*n* = 7), patients who lack knowledge of or comfort with technology (*n* = 4), patients using Palynziq® (pegvaliase-pqpz) (12) (*n* = 2), patients who need labs or exams that cannot be otherwise coordinated (*n* = 2), and children in foster care (*n* = 1).

**Table 2:** Responses by RDs who are currently using telemedicine to provide MNT to patients with IMDs (*n* = 96).

**Question**	**Responses**	**Frequency (%)**
Where are your patients during telemedicine sessions? (Check all that apply)	At their home	93 (96.9%)
	At a partnering clinic	8 (8.3%)
	At their school	1 (1.0%)
	At a health department clinic	6 (6.2%)
	In a hospital	6 (6.2%)
	Other	9 (9.4%)
What type of patients with IMDs do you see via telemedicine? (check all that apply)	Patients with positive results on newborn screening	44 (45.8%)
	Newly diagnosed patients other than newborn screening	38 (40.6%)
	Hospitalized patients seeking nutrition counseling	11 (11.4%)
	Follow-up patients	95 (99.0%)
	Research participants	10 (10.4%)
	Other	5 (5.2%)
Are there patients that cannot be seen via telemedicine? *(1 missing response)*	Yes	65 (68.4%)
	No	30 (31.6%)
What type of patients cannot be seen via telemedicine? (check all that apply) *(66 respondents)*	Newly diagnosed patients	30 (45.5%)
	Hospitalized patients	22 (33.3%)
	Patients out of state	15 (22.7%)
	Other	36 (54.5%)
Do you bill for telemedicine services?	Yes	48 (50.0%)
	No	34 (35.4%)
	I don't know	14 (14.6%)
What is your reimbursement rate?	N/A	48
	I don't know	42 (87.5%)
	<50%	2 (4.2%)
	50% or more	4 (8.3%)
Do you see patients via telemedicine independently or under the supervision of another provider? *(1 missing response)*	Independently	38 (40.0%)
	Under supervision of another provider	57 (60.0%)
Do you bill independently or under supervising provider (such as an MD or NP)?	N/A	37
	Not billing, covered by grant or contract	12 (20.3%)
	Billing independently	6 (10.2%)
	Billing under supervising provider	36 (61.0%)
	I don't know	5 (8.5%)
Where do telemedicine patients have blood draws? (Check all that apply)	Originating site lab	45 (46.9%)
	Local lab	84 (87.5%)
	Primary care provider office	36 (37.5%)
	Other	35 (36.5%)
Are you satisfied with the use of telemedicine for patients with inherited metabolic disorders?	Yes, very satisfied	36 (37.5%)
	Yes, somewhat satisfied	57 (59.4%)
	No, not satisfied	3 (3.1%)

Patients seen via telemedicine who required a blood draw for monitoring received phlebotomy services at the following locations: the originating site (46.9%), a local laboratory (87.5%), their primary care provider's office (37.5%), and other (36.5%). Other locations included home-based bloodspots on filter papers (*n* = 22), at clinic or hospital (*n* = 4), and home-based blood draws (*n* = 1).

Regarding RD satisfaction with telemedicine services for patients with IMDs, 59.4% of RDs reported that they were somewhat satisfied, 37.5% reported that they were very satisfied, and 3.1% reported that they were dissatisfied with virtual visits. The main reason for dissatisfaction with telemedicine was suboptimal communication with patients/families compared to in-person visits (*n* = 3).

Seventy-eight of the RD respondents indicated a need related to improving implementation of telemedicine, including staff training, patient education materials, and provider education materials. Two patient education materials that were specifically requested were resources for patients on how to install and use telemedicine platforms and videos on how to mix formula (medical food). Other needs identified were tips on conducting a nutrition-focused physical exam via telemedicine, improved patient access via primary care providers, higher quality equipment, and interpreter services.

### Changes Made to Clinical Care

“*The COVID-19 emergency response has forced us to think differently about how we provide care to patients. We now offer care in a variety of ways instead of relying solely on in-person appointments.” – RD survey respondent*

Ninety-seven (82.9%) RD participants responded to the survey question requesting a list of three changes made to clinical care for patients with IMDs in response to the COVID-19 pandemic. Changes to clinical care were also mentioned by 29 RDs in the additional comments. The themes related to changes made in clinical practice, along with the number of times each theme was mentioned, are:

#### Telemedicine (*n* = 77)

Virtual visits were a common response to the COVID-19 pandemic. RDs reported incorporating a variety of telemedicine platforms and applications. Some institutions have changed policies to allow patients to be seen from home instead of from a satellite clinic. RDs described successful implementation of telemedicine for MNT for patients with IMDs. Five RDs expressed plans to continue with telemedicine services after the COVID-19 pandemic is resolved.

#### Monitoring (*n* = 56)

RDs are providing MNT for patients using fewer blood draws for laboratory assessments and/or increasing reliance on local laboratories for blood draws. Some reported use of home visiting nurses to obtain blood samples. There is an increase in the number and types of patients monitored with home bloodspot filter papers.

#### Patient Interactions Outside of Telemedicine (*n* = 44)

There is an increased use of phone, mail, and email to interact with patients. RDs reported having more frequent communications with patients and providing more social support. Increased time is spent helping patients access medical foods due to loss of employment or changes in health insurance.

#### Nutrition Management (*n* = 43)

RDs reported that they are extending prescriptions without visits and without recent laboratory results, making fewer changes to diets, delaying weaning toddlers off of formula, shipping medical foods and modified low-protein foods directly to patients, and asking patients to stock up on medical foods. Patients were asked to maintain a 1- to 2-month supply of medical foods and modified low-protein foods, but not to hoard these products as supplies appeared to be limited.

#### Telecommuting (*n* = 29)

RDs reported that they are working from home and that there is an increased use of virtual meetings and rounds with fellow staff members.

#### Decreasing Volume of Patients Seen by Clinic (*n* = 17)

RDs reported that they have canceled non-urgent visits and have ceased non-COVID-19 research activities. Concern was expressed related to patient needs that may be unmet during this time and that postponing some clinical care activities will create future needs.

#### Other Technology Use (*n* = 11)

There is an increased use of electronic medical records and decreased use of paper records, as well as use of technology for group social activities.

#### Scope of Work (*n* = 11)

There have been changes in duties due to redeployment of staff and due to increased need for administrative work. RDs are spending more time helping patients access medical food due to changes in employment and insurance status. Redeployment of genetic metabolic RDs and other clinic staff members to other clinics in the hospital may temporarily lead to decreased resources for patients with IMDs.

#### Developing Emergency Plans (*n* = 6)

RDs reported that they are working to ensure that all patients have up-to-date emergency letters and sick day plans, and reinforcing the need to contact the treating physician if a patient with an IMD becomes sick.

### Benefits of Telemedicine

In addition to the barriers and strategies listed above, analysis of the additional comments section led to the identification of benefits related to telemedicine:

#### Benefits (*n* = 17)

RDs are able to see patients that previously would not come to clinic due to travel burdens and patients benefit from decreased travel time. Other benefits to RDs include increased communication with teenage patients as they talk more on telemedicine visits than in-person and increased insight into how patients/families manage their disorders at home. In addition, when discussing food-related questions, patients can show the RD the food item they are asking about.

## Discussion

The World Health Organization declared COVID-19 to be a pandemic on March 11, 2020 (13). In the United States, the first statewide stay-at-home order was put in place on March 19, 2020 (14). This survey was conducted April 30 through May 13, 2020. During this time, RDs reported a large increase in the number of patients seen via telemedicine in response to the COVID-19 pandemic, along with increased use of telephone and email communications. In addition, a variety of strategies were implemented in response to the COVID-19 pandemic, including changes to monitoring and nutrition management practices.

Telemedicine has become the predominant form of providing MNT for patients with IMDs during the COVID-19 pandemic. Most RDs reported that their institution provides healthcare using telemedicine in general, while 6.5% reported that their institution does not provide such services. Approximately one-quarter of RDs reported that they had implemented telemedicine for MNT for patients with IMDs prior to the onset of the COVID-19 pandemic, while two-thirds reported that they implemented telemedicine after the onset of the pandemic. Four RDs reported no plans to implement telemedicine for patient care and instead rely on telephone communication. Consistent with the barriers identified in the qualitative analysis, these four reported institutional and patient barriers, as well as limitations associated with telemedicine.

We identified several barriers to the implementation of telemedicine for MNT for patients with IMDs. Limitations of virtual visits was the most commonly mentioned barrier and included the inability to obtain accurate anthropometric measurements and the inability to obtain blood samples for monitoring. Strategies to address these barriers included providing accurate home scales to patients, relying on home visiting nurses or local laboratories for blood draws, and increasing the number of patients using filter papers for blood monitoring. Language barriers and the inability to have an interpreter join the telemedicine visit were also identified as barriers. Some platforms do not allow an additional provider, such as an interpreter, to join the virtual visit. The need for interpreters, and sometimes for both RD and MD to meet with the patient at the same time, makes it vital that institutions consider this need when choosing a telemedicine platform and creating institutional policies about telemedicine.

Patient access to technology was the second most commonly mentioned barrier and included lack of internet access and lack of access to smartphone or appropriate computer equipment. In a 2019 survey by the Pew Research Center, 81% of Americans had a smartphone overall, but only 71% in rural areas (15). Without a smartphone, a computer with internet access, camera, microphone, and speaker is required. Internet access in rural areas may be limited, as only 63% of rural Americans have broadband and 69% have a desktop computer or laptop (15). Access to adequate technology and internet service is also affected by socioeconomic status (16) and demographic characteristics such as age and education (17). Smartphone or computer and internet access also is a problem in other countries and is impacted by age, education, and income (18).

Libraries can assist by providing access to appropriate computer equipment and internet in a private location. Some primary care providers also may provide access to equipment for patients. Assistance programs may also provide data plans for smartphones, where funds are available. Another potential way to address patient knowledge and access barriers is the use of community health workers who can assist the family with telemedicine. Social distancing and access to personal protective equipment would need to be taken into consideration for this strategy.

Using telemedicine can be time-consuming, including training of staff, scheduling of patients, and preparing patients for the visit. This increase in workload comes at a time when clinic staff may be redeployed to assist with COVID-19 patients. As a result, RDs report that metabolic clinics are limiting non-urgent visits and extending prescriptions without visits. Limiting visits may result in delayed care with unknown long-term implications.

Telemedicine implementation during COVID-19 was facilitated in the US by relaxation in Centers for Medicaid and Medicare Services (CMS) regulations about circumstances in which a healthcare provider can bill for telemedicine (19, 20). Even though RDs reported being highly satisfied with virtual visits, it is not clear how sustainable telemedicine will be if CMS rules return to pre-COVID-19 status after the pandemic is over.

Patient education materials were identified as an unmet need by approximately three-quarters of respondents. Patient education materials should focus on increasing patient comfort with telemedicine in general and providing specifics of how to use platforms. Language barriers need to be addressed by providing patient education materials in multiple languages, but clinics do not always have the staff or funds necessary to translate materials into the needed languages. Some RDs reported that patients experienced distractions in their home environment or that patients were “multi-tasking” during telemedicine appointments. Patient education materials may help patients to better understand their roles and responsibilities in the success of the telemedicine visit.

Widespread disruptions to clinical care for patients with IMDs have been previously reported, such as in the aftermath of hurricanes Katrina and Rita in the United States. Recommendations that arose in response to these disruptions included the development of emergency plans for communication, identification of back-up laboratories, development of plans for continued access to medical foods, and preparation of emergency patient instructions (21). However, prior to the COVID-19 pandemic, few institutions had emergency plans for the use of telemedicine or for alternate means of obtaining blood samples during natural disasters or other public health emergencies.

While this survey focused on RDs' experiences with telemedicine, future research should include the experiences of patients with telemedicine to identify barriers and unmet needs from their point of view. The purpose of this survey was to identify barriers and the strategies that RDs use to overcome them. We did not ask specifically about the benefits of telemedicine, although several respondents volunteered this information in their additional comments. Benefits of telemedicine to both RDs and patients is an area that requires more in-depth research. As indicated by the comments, patients who are not able to come clinic for in-person visits due to distance or other barriers may be engaged by telemedicine. However, the impact of limiting non-urgent visits and extending prescriptions without in-person exams or accurate anthropometrics is not known at this time. Future research also is needed to assess the impact of telemedicine visits on patient outcomes. Telemedicine may have both positive and negative impacts on patients. This survey was conducted early in the pandemic, at a time when RDs were still adjusting to the shift to telemedicine visits. Additional strategies to address barriers may have been developed since that time, which could be captured by follow-up studies.

In conclusion, telemedicine has played a major role in the provision of MNT for patients with IMDs during the COVID-19 pandemic. Telemedicine for patients with IMDs has been generally well-accepted by both patients and genetic metabolic RDs. Despite limitations, comments revealed significant support for telemedicine effectiveness and its potential to improve access to care, including people living in rural areas, those without transportation, and those unable to take time off work. By allowing patients to receive services while remaining at home, telemedicine decreases the risk of exposure to the SARS-CoV-2 virus. However, creative strategies are needed to address remaining barriers and unmet needs so that MNT telemedicine services are optimized for patient care, improved outcomes, and sustainability post pandemic.

## Data Availability Statement

The raw data supporting the conclusions of this article will be made available by the authors, without undue reservation.

## Ethics Statement

The studies involving human participants were reviewed and approved by Emory University Institutional Review Board. Written informed consent for participation was not required for this study in accordance with the national legislation and the institutional requirements.

## Author Contributions

RS: conceptualization and funding acquisition. AK: data curation and writing—original draft. TP and AK: formal analysis and project administration. RS, TP, and AK: methodology and writing—review & editing. All authors gave final approval of the version to be published and meet the ICMJE criteria for authorship.

## Conflict of Interest

The authors declare that the research was conducted in the absence of any commercial or financial relationships that could be construed as a potential conflict of interest.
